# Decoding competitive endogenous RNA regulatory network in postoperative cognitive dysfunction

**DOI:** 10.3389/fnins.2022.972918

**Published:** 2022-09-20

**Authors:** Wei Wang, Pengwei Huo, Lei Zhang, Gang Lv, Zhongyuan Xia

**Affiliations:** ^1^Department of Anesthesiology, Renmin Hospital of Wuhan University, Wuhan, China; ^2^Department of Anesthesiology, Yulin No.2 Hospital, Yulin, China

**Keywords:** postoperative cognitive dysfunction, competitive endogenous RNA network, circRNA, Wnt signaling, bioinformatic analysis

## Abstract

Postoperative cognitive dysfunction (POCD) is a common postoperative neurological complication in elderly patients. Circular RNAs (circRNAs) are abundant in the mammalian brain and can probably regulate cognitive function. However, the competitive endogenous RNA (ceRNA) regulatory network in POCD remains illiterate. Transcriptomic signatures in the hippocampus of POCD mice derived from the Gene Expression Omnibus (GEO) dataset GSE190880, GSE95070, and GSE115440 were used to identify the circRNA, miRNA, and mRNA expression profiles of POCD mice compared with controls, respectively. A set of differentially expressed RNAs, including 119 circRNAs, 33 miRNAs, and 49 mRNAs were identified. Transcript validation showed the enhanced expression of circ_0001634, circ_0001345, and circ_0001493. A ceRNA regulatory network composed of three circRNAs, three miRNAs, and six mRNAs was established. The hub mRNAs in the ceRNA network were further found to be involved in the hormone catabolic process and regulation of canonical Wnt signaling pathway, revealing their crucial role in POCD. Finally, three miRNAs and four mRNAs were verified by qRT-PCR. These results based on bioinformatics and PCR array suggest that circ_0001634/miR-490-5p/Rbm47, circ_0001634/miR-490-5p/Sostdc1, circ_0001634/miR-7001-5p/Sostdc1, circ_0001345/miR-7001-5p/Sostdc1, and circ_0001493/miR-7001-5p/Sostdc1 may be novel diagnostic biomarkers and therapeutic targets for POCD.

## Introduction

Postoperative cognitive dysfunction (POCD) is a neurocognitive disorder such as acute or persistent impairments in attention, learning, memory, and information processing that occurs predominantly in geriatric patients who undergo anesthesia and major surgery (Suwanabol et al., 2022). The long-term impact of POCD is associated with a worse overall quality of life, increased mortality, and a heavy burden on society and families (Boone et al., 2020; Tang et al., 2020). Recent evidence suggests that various elements, such as aberrant expression of apolipoprotein E4 genotype, blood-brain barrier (BBB) compromise, surgery-induced neuroinflammation, microglial activation, mitophagy impairment, and iron accumulation (McDonagh et al., 2010; Chen et al., 2020; Danielson et al., 2020; Wu et al., 2020; Yang et al., 2020), are intimately involved in all stages of postoperative cognitive decline. Despite major advances in comprehending pathogenesis and prevention methods, the overall curative effect remains inadequate. Consequently, it is imperative to screen promising therapeutic targets for POCD.

Circular RNA (circRNA), unlike conventional linear RNA, is a subtype of endogenous non-coding RNAs with covalently closed-loop structures that confer high resistance to degradation (Chen, 2020). CircRNAs have been proposed to modulate gene transcription, to act as microRNA (miRNA) or RNA-binding protein (RBP) decoys, and to function as protein scaffolds (Kristensen et al., 2019). However, the overwhelming majority of circRNAs are thought to act as competing endogenous RNAs (ceRNAs), which specify that circRNAs can compete with mRNAs for binding to the shared miRNAs and thereby indirectly modify gene expression at the post-transcriptional level (Chen, 2020). The abundance of circRNAs in the mammalian brain, particularly synaptoneurosomes, is highlighted by RNA sequencing data (Rybak-Wolf et al., 2015). Mounting evidence also suggests circRNAs play a vital role in cognitive impairments such as Alzheimer impisease (AD) (Dube et al., 2019). Downregulation of circCwc27 improved cognitive capacity in the AD mouse (Song et al., 2022). Knockdown of circTshz2-2 alleviated obesity-induced spatial memory decline via modulating BDNF/TrkB signaling pathway (Yoon et al., 2021). CircPtk2 contributed to sepsis-provoked cognitive dysfunction by serving as a sponge of miR-181c-5p to facilitate HMGB1 expression (Li et al., 2021). Niu et al. (2021) proposed that aerobic exercise mitigated vascular cognitive impairment by activating circRIMS2/miR-186/BDNF axis. Additionally, plasma circRNA-089763 is positively correlated with the occurrence of POCD (Wang et al., 2019; Zhou et al., 2020). A microarray analysis screened 210 differentially expressed circRNAs in POCD patients’ serum, such as circCPNE1, circUBE3B, and circITSN1 (Gao et al., 2020). Another microarray profiling highlighted three circRNAs (circ_22058, circ_44122, and circ_22673) as key elements in POCD (Wu et al., 2021). Circ_009789- and circ_004229-associated ceRNA networks were identified to elucidate the mechanism underlying susceptibility to POCD in aged mice (Zhang et al., 2022). CircShank3 may be involved in dexmedetomidine-mediated protection against POCD via targeting the p53 and NF-κB signaling pathways (Cao et al., 2020). Nevertheless, the role of circRNAs in POCD remains elusive.

MiRNAs are small (18–22 nucleotide long) non-coding RNAs that trigger post-transcriptional repression of gene expression by directly binding to the 3′-untranslated region (UTR) of mRNAs, profoundly governing neurodevelopment and neurodegeneration (Singh and Yadav, 2020). Multiple aberrantly expressed miRNAs, such as miR-124, miR-146a, and miR-381, were implicated in the development of POCD (Chen et al., 2019a,b; Wang et al., 2021). However, the underlying mechanisms of miRNAs in the neuropathogenesis of POCD are still unclear.

**FIGURE 1:**
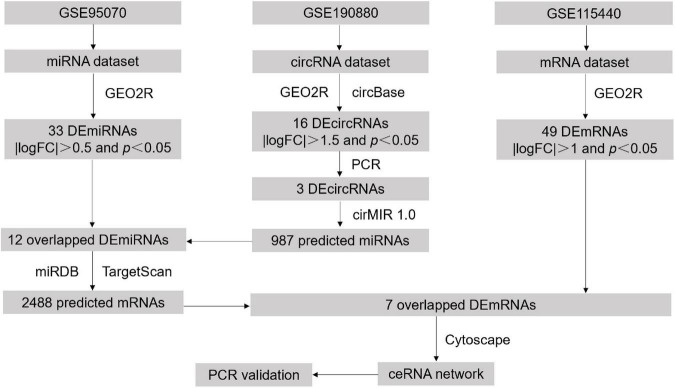
Workflow of the study design.

In this study, we extracted the differentially expressed circRNAs, miRNAs, and mRNAs in POCD from Gene Expression Omnibus (GEO) databases. According to the flowchart diagram (Figure 1), circRNA-miRNA pairs and miRNA-mRNA pairs were successively identified for constructing a ceRNA regulatory network. Furthermore, functional enrichment analysis and transcript validation were implemented to identify the hub genes and interpret potential regulatory mechanisms in the development of POCD. The present findings may strengthen our understanding of POCD.

## Materials and methods

### Expression microarray data

The circRNA dataset (GSE190880) was retrieved from the GPL21826 platform and contained 3 pairs of hippocampus from POCD and control mice (Ran et al., 2022). The miRNA dataset (GSE95070) was retrieved from the GPL19117 platform and contained 5 pairs of hippocampus from POCD and control mice (Wei et al., 2017). Meanwhile, we also downloaded the mRNA expression profiles from GSE115440 (GPL11533) (Yang et al., 2019), including 3 pairs of hippocampus tissues from POCD and control mice, to further construct the ceRNA network about POCD. The profiles of these three microarray datasets (GSE190880, GSE95070, and GSE115440) regarding circRNAs, miRNAs, and mRNAs were listed in Table 1. In these three microarray datasets, aseptic tibia fracture in C57BL/6 male mice was applied to mimic POCD.

**TABLE 1:** The profiles of three microarray datasets from the GEO database.

Data source	Series	Platform	Author	Year	Country	Sample (POCD/control)
circRNA	GSE190880	GPL21826	Ran	2021	China	3/3
miRNA	GSE95070	GPL19117	Wei	2017	China	5/5
mRNA	GSE115440	GPL11533	Mkrtchian	2018	Sweden	3/3

### Identification of the differentially expressed circular RNAs, microRNAs, and mRNAs

Differentially expressed (DE) analysis between POCD and control hippocampus samples were performed using GEO2R (Barrett et al., 2013), an online program based on R language that can analyze any GEO series to compare two groups of data. The cut-off standard of DEcircRNAs was set to *p* < 0.05 and |logFC| > 1.5, the cut-off standard of DEmiRNAs was set to *p* < 0.05 and |logFC| > 0.5, and the cut-off standard of DEmRNAs was set to *p* < 0.05 and |logFC| > 1. Principal component analysis (PCA) and heatmap (linkage method: row clustering; distance measure: euclidean) were visualized using the Xiantao search tool.^1^

### Establishment of circular RNAs– microRNA–mRNA network

The circRNA identification (ID) of DEcircRNAs were first converted into the circRNA ID in the circBase (Glažar et al., 2014),^2^ an online database that integrates thousands of public circRNAs across five species and their genomic profiles. Only genomic sequences in FASTA format of annotated circRNAs were further analyzed using the circMIR1.0 software, a forecasting instrument of circRNA-miRNA interactions based on miRanda and RNAhybrid that can visualize the binding sites of miRNA adsorption. Overlapping miRNAs between the target miRNAs and DEmiRNAs screened in the GSE95070 were considered candidate miRNAs. Moreover, miRDB (Chen and Wang, 2020)^3^ and Targetscan (McGeary et al., 2019) (version 8.0)^4^ databases were used to predict the target mRNAs of the candidate miRNAs. The Venn diagram drawn in the Xiantao search tool was utilized to overlap the miRNA-forecasted mRNAs and DEmRNAs in the GSE115440. Finally, the ceRNA network was constructed using Cytoscape (Otasek et al., 2019) (version 3.9.1), by integrating circRNA-miRNA pairs and miRNA-mRNA pairs.

### Functional classifications and pathway enrichment analysis

The Gene Ontology (GO) project is an ontological annotation resource that describes gene product function based on multiple databases (Ashburner et al., 2000). Kyoto Encyclopedia of Genes and Genomes (KEGG)^5^ is an integrated tool that provides a new perspective on molecular-level functions, diseases and drugs (Kanehisa et al., 2016). The DAVID Knowledgebase^6^ is a bioinformatics data resource based on a single-linkage method for the agglomeration of millions of genes that can be entered into DAVID gene clusters (Huang da et al., 2009a,b). GO annotation and KEGG pathway enrichment analysis were implemented using the Xiantao search tool. A *p*-value of <0.05 was set as the cut-off point.

### Animals

Twelve-month-old C57BL/6 male mice weighing 25–35 g were purchased from Hubei Provincial Center for Disease Control and Prevention. Mice with *ad libitum* access to food and water were housed in a colony room with a temperature of 22–25°C, a humidity of 50%, and a 12-h light/dark cycle. The animals were acclimated to the environment for 7 days before experimental manipulation. All experimental procedures were approved by the Laboratory Animal Ethics Committee in Renmin Hospital of Wuhan University (No. WDRM-20210709).

### Animal model of postoperative cognitive dysfunction

The animals were randomly assigned to two groups: the POCD group (*n* = 6) and control group (*n* = 6). Intramedullary fixation for open tibial fracture under isoflurane anesthesia has been widely used as a POCD model (Yang et al., 2019). Briefly, mice were exposed to 3% isoflurane for the anesthesia induction, followed by 1.5% isoflurane in 100% oxygen for the maintenance. After shaving and disinfecting the animal, a skin incision was made on the lateral tibia, and a 0.3-mm intramedullary fixation pin was inserted into the medullary cavity. Next, an osteotomy was performed at the middle and distal third of the bone, and the wound was closed with 5-0 Vicryl suture (Ethicon, Somerville, NJ, USA). The entire procedure from the induction to the end of surgery lasted 30 min. A warm pad was utilized to keep the body temperature around 37°C throughout the surgery. Then, 2% lidocaine solution and 1% tetracaine hydrochloride mucilage was applied locally for postoperative analgesia twice daily until 3 days after surgery. The control group received 100% oxygen without surgery or anesthesia.

### Open field test

The open field test (OFT) was employed to assess the locomotor activity of the mice 3 days after the surgery. The apparatus was an opaque plastic cube box with a side length of 45 cm. Each mouse was gently placed in the central area and left to move freely for 5 min. The total distance and time spent in the center area were recorded using SuperMaze software (XinRuan Information Technology, Shanghai, China).

### Morris water maze

Spatial learning and memory was assessed using Morris water maze test 4 days after the surgery. The apparatus was a circular plastic pool of 120-cm diameter and 50-cm height surrounded by four curtains. The pool was equipped with a 6-cm-diameter hidden platform placed in the center of the fourth quadrant. Prior to the experiment, the pool was filled with opaque water at around 25°C to a depth of about 30 cm, approximately 1 cm above the platform. Each mouse received four daily trials with a 30-min intertrial interval for four consecutive days to find the platform, and was randomly placed at one of the four quadrants. Mice that failed to find the platform within 90 s were manually directed to the platform. The escape latency (time taken to reach the platform) in the four quadrants on the same day was averaged. The platform was removed on the fifth day to allow for probe trial. During the 90-s session, the total time spent in target quadrant and the frequency of crossing over the target quadrant were recorded. The swimming trajectory of the animals were recorded automatically via a video tracking system (XinRuan Information Technology, Shanghai, China).

### RNA extraction and quantitative real-time polymerase chain reaction

Firstly, five circRNAs (circ_0001634, circ_0001345, circ_0001493, circ_0000487, and circ_0001468) whose host genes may be related to cognitive function were selected for quantitative real-time polymerase chain reaction (qRT-PCR). To verify the reliability and accuracy of the predicted ceRNA network, three miRNAs (miR-6912-5p, miR-490-5p, and miR-7001-5p), and four mRNAs (Rbm47, Sostdc1, Cdh3, and Sfrp5) were selected for qRT-PCR. Total RNA was extracted from the hippocampus tissue of the control and POCD group using TRIpure reagent (ELK, Wuhan, China). For circRNAs and mRNAs, cDNA was generated from template RNA using EntiLink™ Reverse Transcriptase Kit (ELK, Wuhan, China), and analyzed with EnTurbo™ SYBR Green PCR SuperMix (ELK, Wuhan, China) using StepOne™ Real-Time PCR system (Life Technologies, Carlsbad, CA, USA). The reaction procedure was as follows: 95°C for 1 min, followed by 40 cycles of 95°C for 15 s, 58°C for 20 s and 72°C for 45 s. For miRNAs, cDNA was generated using EntiLink™ 1st Strand cDNA Synthesis Kit (ELK, Wuhan, China), and analyzed with EnTurbo™ SYBR Green PCR SuperMix (ELK, Wuhan, China) using QuantStudio 6 Flex real-time PCR system (Life Technologies, Carlsbad, CA, USA). The reaction procedure was as follows: 95°C for 30 s, followed by 95°C for 10 s, 58°C for 30 s, and 72°C for 30 s. The relative expression levels of these RNAs were calculated via the 2^–ΔΔ^*^Ct^* method. GAPDH was used as an internal control gene for circRNA and mRNA expression, while U6 was used as an internal control gene for miRNA expression. The primers for these RNAs are shown as Table 2.

**TABLE 2:** The primer sequences used for qRT-PCR.

	Sense	Antisense
circ_0001634	CATGAGCAGTTTTCCTTCCCAG	GGAGAGTGAGGTCACTAGAAACAG
circ_0001345	GACCAAGAGACTGGACGAGT	GGAGAGCTTATTGTCAGAGTGTACA
circ_0001493	CAGCAAGCAGACATACCAC	GTGCTCGTAGTGGTCTGGAC
circ_0000487	GTGTTCTGACAAAACACCTGAGG	CTGTAATGGTGTCCAGGCAGTAAC
circ_0001468	GCTGACCTCAAACCAGAAAACAT	GTTTCTTCACACTACAGAAGGCA
miR-6912-5p	GGCTACAGGGAGGGTGCT	CTCAACTGGTGTCGTGGAGTC
miR-490-5p	GGCCCCATGGATCTCCA	CTCAACTGGTGTCGTGGAGTC
miR-7001-5p	GGAGGCAGGGTGTGAGC	CTCAACTGGTGTCGTGGAGTC
Rbm47	ACCCAGCTACGTGTACTCCTGT	GTTCATATCCTTTCTCCTGCTG
Sostdc1	CATTTCAGTAGCACTGGACTGG	GCTCCAGTACTTTGTTCCATAGC
Cdh3	ATCAGCTCAAATCTAATAAGGACAG	CCATAAAGCTCGTACTTGACAATCT
Sfrp5	GGGACCGAAAGTTGATTGG	TGAATTTGACTGCAAACTTCATC
GAPDH	TGAAGGGTGGAGCCAAAAG	AGTCTTCTGGGTGGCAGTGAT
U6	CTCGCTTCGGCAGCACAT	AACGCTTCACGAATTTGCGT

### Statistical analysis

Quantitative data were presented as the mean ± standard deviation (SD) or median (range). GraphPad Prism version 9.0 (GraphPad Software Inc., San Diego, California, USA) was used for statistical analyses. The statistical significance between the two groups was determined using Student’s *t*-test or non-parametric test. A *p*-value of <0.05 was considered statistically significant.

## Results

### Isoflurane plus orthopedic surgery caused postoperative cognitive dysfunction in mice

**FIGURE 2:**
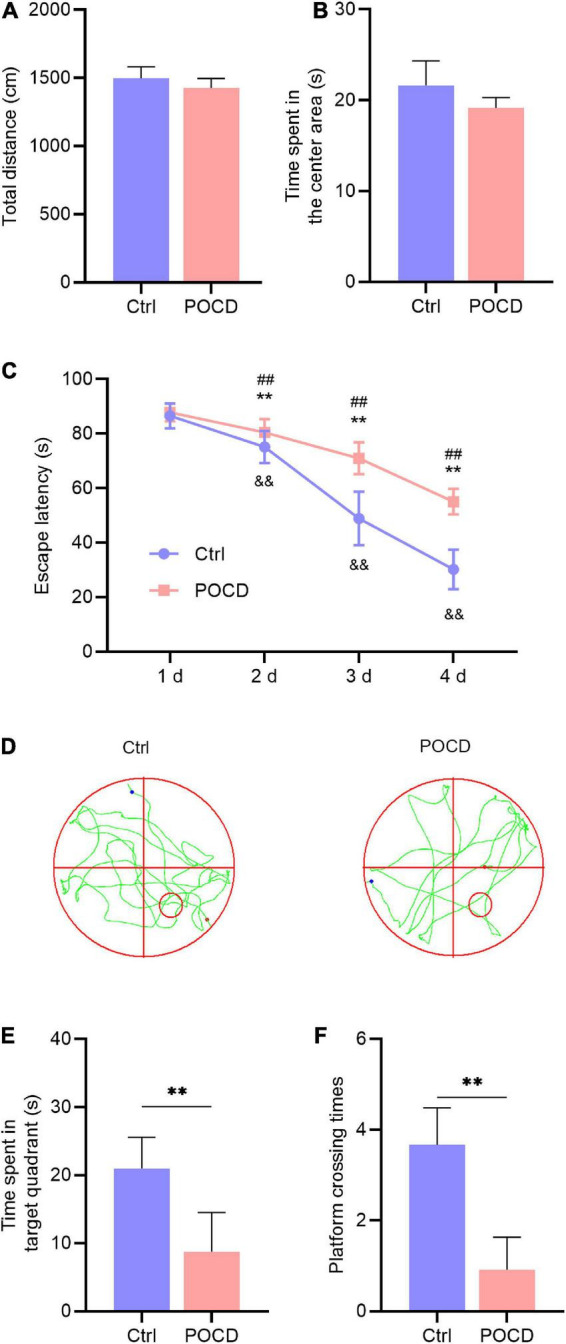
Anesthesia/surgery impaired cognitive function in aged mice (*n* = 6 per group). **(A)** The total distance in the OFT. **(B)** Time spent in the center area in the OFT. **(C)** Escape latency to reach the hidden platform during the 4-day training. Unpaired *t*-test, **p < 0.01 compared to the control group; ^##^*p* < 0.01 compared to the first day in the control group; ^&⁣&^*p* < 0.01 compared to the first day in the POCD group. **(D)** The swimming trajectory of the control and POCD mice during the probe test. The red circle indicates the hidden platform, the red and blue dot indicate the start and end of swimming, respectively. **(E)** Time spent in the target quadrant during the probe test. Unpaired *t*-test, **p < 0.01 compared to the control group. **(F)** Platform crossing times during the probe test. Unpaired *t*-test, **p < 0.01 compared to the control group.

The open feld test showed no statistical difference in the total distance and duration of the central area between the two groups, indicating that anesthesia/surgery did not affect the locomotor activity of the mice (Figures 2A,B). To address whether isoflurane/surgery impaired cognitive function in mice, we performed Morris water maze to assess learning and memory following orthopedic surgery. During hidden platform test, the mice undergoing anesthesia/surgery showed significantly longer escape latency on day 2, 3, and 4 than the untreated control (Figure 2C). During the probe test, time spent in target quadrant and platform crossing times in the mice undergoing anesthesia/surgery were less than those in the control group (Figures 2D–F). These results suggested that isoflurane plus orthopedic surgery caused POCD.

### Data preprocessing

**FIGURE 3:**
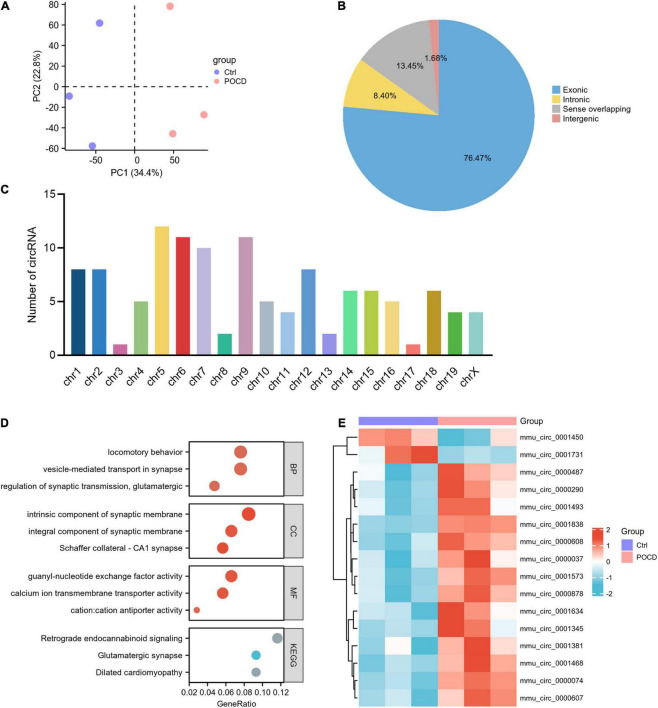
Characteristics of differentially expressed circRNAs (DEcircRNAs) in postoperative cognitive dysfunction (POCD). **(A)** Principal component analysis (PCA) of circRNA expression between POCD and control groups. **(B)** Genomic origin of the DEcircRNAs. **(C)** Chromosomal distribution of the DEcircRNAs. **(D)** Functional classifications and pathway enrichment analysis (GO and KEGG) of the host genes of DEcircRNAs. The horizontal axis denotes the proportion of the host genes in each cluster, and the vertical axis denotes biological process (BP), cellular component (CC), molecular function (MF), and KEGG pathway, respectively. **(E)** Heatmap plots of the 16 circRNAs annotated by circBase.

**FIGURE 4:**
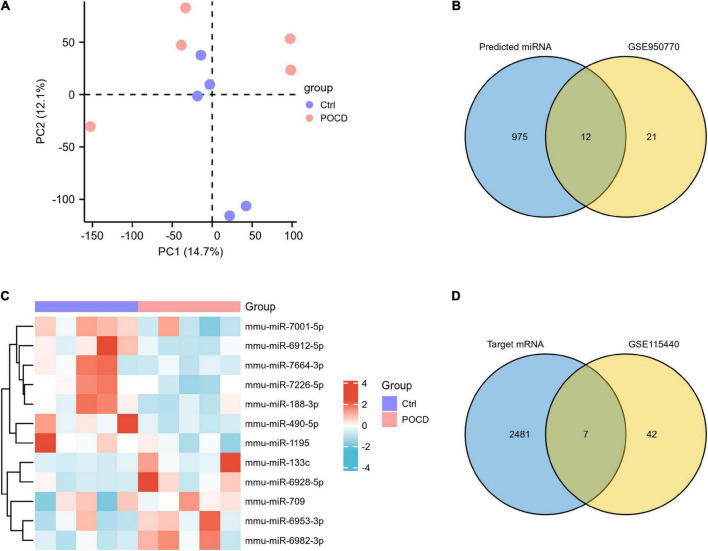
Characteristics of differentially expressed miRNAs (DEmiRNAs) in postoperative cognitive dysfunction (POCD). **(A)** Principal component analysis (PCA) of miRNA expression between POCD and control groups. **(B)** The 12 DEmiRNAs were obtained by overlapping the 987 target miRNAs binding to the three DEcircRNAs and the 33 DEmiRNAs identified in GSE95070. **(C)** The heatmap of the 12 overlapped DEmiRNAs. **(D)** The seven DEmRNAs were obtained by overlapping the 2,488 target mRNAs binding to the 12 DEmiRNAs and the 49 DEmRNAs identified in GSE95070.

The accuracy and reliability of microarray data were evaluated using PCA. By comparing the distribution patterns of all identified circRNAs (Figure 3A) and miRNAs (Figure 4A), we could completely separate POCD samples from control hippocampus.

### Circular RNA expression profiles in postoperative cognitive dysfunction

In the GSE190880 dataset, a total of 119 DEcircRNAs with thresholds of |logFC| > 1.5 and *p* < 0.05 were detected in the hippocampus of POCD mice, of which 103 were up-regulated and 16 were down-regulated. Additionally, 76.47% of DEcircRNAs originated from exonic regions, while 8.40% originated from intronic regions. Sense-overlapping and intergenic circRNAs accounted for 13.45 and 1.68%, respectively (Figure 3B). Chromosomal distribution demonstrated that DEcircRNAs are highly abundant in chr1-19 and chrX, but absent in chr20 and chrY (Figure 3C).

GO and KEGG pathway analyses were used to categorize and annotate the host genes of DEcircRNAs in order to further characterize these in POCD. The enriched GO terms were mainly associated with locomotory behavior (gene ratio = 8/105, *p* = 2.06E-05), vesicle-mediated transport in synapse (gene ratio = 8/105, *p* = 1.64E-05), regulation of synaptic transmission, glutamatergic (gene ratio = 5/105, *p* = 2.63E-05), intrinsic component of synaptic membrane (gene ratio = 9/106, *p* = 1.69E-06), integral component of synaptic membrane (gene ratio = 7/106, *p* = 7.67E-05), Schaffer collateral-CA1 synapse (gene ratio = 6/106, *p* = 1.27E-05), guanyl-nucleotide exchange factor activity (gene ratio = 7/106, *p* = 3.92E-05), calcium ion transmembrane transporter activity (gene ratio = 6/106, *p* = 4.49E-05), and cation:cation antiporter activity (gene ratio = 3/106, *p* = 4.49E-05) (Figure 3D and Supplementary Table 1). Moreover, KEGG pathway enrichment analysis concluded that DEcircRNAs were principally enriched in retrograde endocannabinoid signaling (gene ratio = 5/43, *p* = 0.0007), dilated cardiomyopathy (gene ratio = 4/43, *p* = 0.0010), and glutamatergic synapse (gene ratio = 4/43, *p* = 0.0021) (Figure 3D and Supplementary Table 1). Ulteriorly, the DEcircRNAs were blasted by circBase and 16 annotated circRNAs were identified, of which 14 were up-regulated and 2 were down-regulated, as shown by the hierarchical clustering heatmap (Figure 3E and Table 3).

**TABLE 3:** Differentially expressed circRNAs annotated by circBase in POCD.

circRNA_ID	circBase_ID	Chrom	Strand	Location	Type	Gene	LogFC	*P*-value
circ_011823	circ_0000878	chr18	+	63,755,035–63,760,785	Exonic	Wdr7	1.633	0.009
circ_009299	circ_0000607	chr15	−	68,165,752–68,170,223	Exonic	Zfat	1.543	0.009
circ_007883	circ_0001634	chr7	−	132,759,388–132,779,385	Exonic	Fam53b	1.596	0.032
circ_016800	circ_0000608	chr15	+	69,013,357–69,029,910	exonic	Khdrbs3	1.526	0.005
circ_011539	circ_0000074	chr1	+	127,791,604–127,799,553	Exonic	Ccnt2	1.546	0.001
circ_008286	circ_0000487	chr13	−	89,991,072–90,001,084	Exonic	Xrcc4	1.506	0.028
circ_009389	circ_0001381	chr5	−	106,619,539–106,666,845	Exonic	Zfp644	1.639	0.047
circ_009434	circ_0000037	chr1	−	52,708,163–52,709,755	Exonic	Mfsd6	1.601	0.033
circ_011555	circ_0001345	chr5	−	43,758,221–43,773,659	Exonic	Fbxl5	1.576	0.039
circ_009748	circ_0001838	chr9	−	106,952,311–106,978,774	Exonic	Dock3	1.541	<0.001
circ_017841	circ_0001493	chr6	−	90,689,579–90,694,850	Exonic	Iqsec1	1.506	0.039
circ_016934	circ_0001573	chr7	−	59,479,061–59,481,464	Sense overlapping	Gm22632	2.078	0.007
circ_002179	circ_0001468	chr6	−	38,818,229–38,819,313	Exonic	Hipk2	1.581	0.005
circ_016597	circ_0000290	chr11	+	75,390,071–75,391,227	Exonic	Smyd4	1.560	0.018
circ_009489	circ_0001450	chr6	+	29,372,580–29,372,670	Intronic	Calu	−1.594	0.047
circ_011181	circ_0001731	chr8	−	122,908,667–122,916,045	Exonic	Ankrd11	−1.668	0.035

### Validation of differentially expressed circRNAs

**FIGURE 5:**
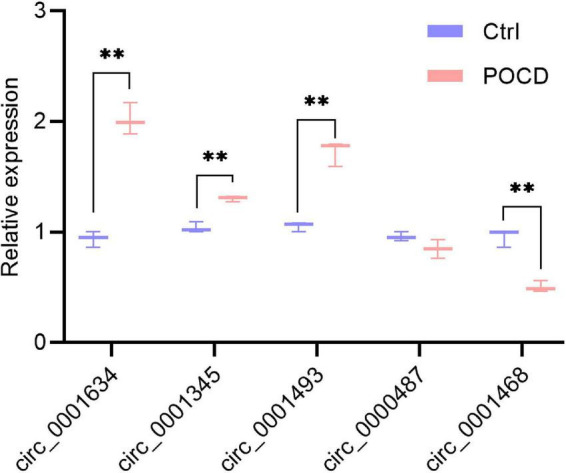
Transcript verification of circRNAs whose host genes are likely to be associated with cognitive function. Target cirRNA expression were normalized to GAPDH expression (ΔCt). Non-parametric test, **p < 0.01 compared to the control group.

To validate the expression of DEcircRNAs, five circRNAs whose host genes are possibly related to cognitive function were selected for verification by qRT-PCR. The expression of mmu_circ_0001634 (circFam53b), mmu_circ_0001345 (circFbxl5), and circ_0001493 (circIqsec1) were found to be upregulated in the hippocampus of POCD mice, which was consistent with the sequencing results. There was no significant difference in the expression of mmu_circ_0000487 (circXrcc4) between the two groups. Contrary to the sequencing result, isoflurane/surgery obviously diminished mmu_circ_0001468 (circHipk2) expression (Figure 5). To unveil insights into the potential functional mechanisms of circFam53b, circFbxl5, and circIqsec1, we predicted 987 miRNAs interacting with circRNAs and the putative binding domains with the aid of circMIR1.0 software (Figure 4B).

### MicroRNA expression profiles in postoperative cognitive dysfunction

A total of 33 significant DEmiRNAs with cut-off criteria of |logFC| > 0.5 and *p* < 0.05 were screened in the hippocampus of POCD mice. We identified 12 overlapping DEmiRNAs by integrating GSE95070 data and predicted results, of which five were upregulated and seven were downregulated (Figures 4B,C and Supplementary Table 2). According to the ceRNA **hypothesis** that circRNAs could compete with mRNAs for the same miRNAs, we predicted the mRNAs downstream of 12 miRNAs. The results reflected that 2,488 target genes of the 12 DEmiRNAs were acquired by the databases of miRDB and TargetScan. Furthermore, a total of 49 significant DEmRNAs were extracted in GSE115440. The Venn diagram revealed that seven mRNAs were shared by the predicted genes of the above-mentioned circRNA-targeted miRNAs and the DEmRNAs from GSE115440 (Figure 4D and Supplementary Table 3).

### Construction of the competitive endogenous RNA regulatory network and experimental verification

**FIGURE 6:**
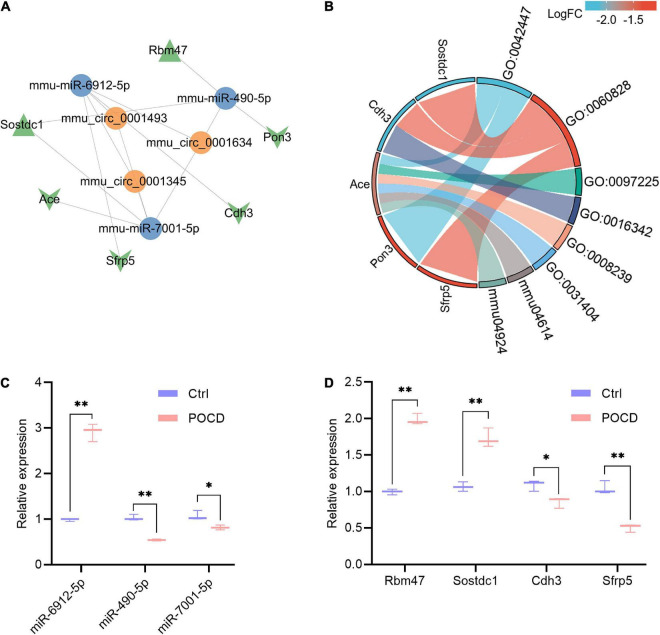
Potential competing endogenous RNA (ceRNA) regulatory network, enrichment analysis of DEmRNAs in the ceRNA network, and qRT-PCR validation of three miRNAs and four mRNAs. **(A)** The ceRNA regulatory network includes three circRNAs, three miRNAs, and six mRNAs. The orange color indicates circRNAs, the blue color indicates miRNAs, and the green color indicates mRNAs (Triangle and V denote upregulation and downregulation, respectively). **(B)** Functional classifications and pathway enrichment analysis (GO and KEGG) of DEmRNAs in the ceRNA network. The right half part indicates enriched biological process (BP), cellular component (CC), molecular function (MF), and KEGG pathway; the left half part indicates the genes involved in the corresponding BP, CC, MF, and pathways. GO:0042447, hormone catabolic process; GO:0060828, regulation of canonical Wnt signaling pathway; GO:0097225, sperm midpiece; GO:0016342, catenin complex; GO:0008239, dipeptidyl-peptidase activity; GO:0031404, chloride ion binding; mmu04614, renin-angiotensin system; mmu04924, renin secretion. **(C,D)** Quantitative RT-PCR validation of miR-6912-5p, miR-490-5p, miR-7001-5p, Rbm47, Sostdc1, Cdh3, and Sfrp5. Target miRNA expression were normalized to U6 expression, and mRNA expression were normalized to GAPDH expression (ΔCt). Non-parametric test, *p < 0.05, **p < 0.01 compared to the control group.

A ceRNA regulatory network based on 3 circRNAs, 12 miRNAs, and 7 mRNAs was created to further investigate the mechanisms by which circRNAs and miRNAs affect the occurrence and development of POCD. Eventually, 10 circRNA-miRNA pairs and 7 miRNA-mRNA pairs were identified, which were composed of three circRNAs, three miRNAs, and six mRNAs (Figure 6A). The enriched GO terms were chiefly associated with the hormone catabolic process, regulation of canonical Wnt signaling pathway, sperm midpiece, catenin complex, dipeptidyl-peptidase activity, and chloride ion binding. Moreover, the enriched KEGG pathway were renin-angiotensin system and renin secretion (Figure 6B and Supplementary Table 4). To substantiate the potential interaction in the ceRNA, these three miRNAs and Wnt-related genes were selected for verification by qRT-PCR. The enhanced expression of miR-6912-5p, Rbm47 and Sostdc1 were observed in the POCD group, while the expression of miR-490-5p, miR-7001-5p, Cdh3, and Sfrp5 were significantly decreased in the POCD group (Figures 6C,D).

## Discussion

In our study, we outlined comprehensive transcriptome profiles of POCD. A total of 119 circRNAs, 33 miRNAs, and 49 mRNAs were identified as differentially expressed. The circRNA-miRNA-mRNA triple regulatory network consisted of three circRNAs, three miRNAs, and six mRNAs. Biological process enrichment analysis revealed that the bulk of DEmRNAs in the ceRNA network were involved in the “hormone catabolic process” and “regulation of canonical Wnt signaling pathway.” Transcript validation suggested that circ_0001634/miR-490-5p/Rbm47, circ_0001634/miR-490-5p/Sostdc1, circ_0001634/miR-7001-5p/Sostdc1, circ_0001345/miR-7001-5p/Sostdc1, and circ_0001493/miR-7001-5p/Sostdc1 axis were likely to participate in the development of POCD.

The vast majority of circRNAs are derived from a single or multiple exons of known coding genes and thereby located in the cytoplasm (Kristensen et al., 2019). Our analysis showed that up to 76.47% DEcircRNAs were from exons, and 14 out of 16 annotated circRNAs were of exon origin, which implied that circRNAs probably function as competing endogenous RNAs in the pathogenesis of POCD because the ceRNA network merely exists in the cytoplasm (Chen, 2020). Enrichment analysis showed the corresponding parental or host genes of DEcircRNAs were implicated in synaptic plasticity and mainly located in the Schaffer collateral-CA1 synapse, suggesting circRNAs are extensively involved in the regulation of cognitive function.

Circ_0001493, circ_0000487, circ_0001468, and circ_0001345 are spliced from IQ motif and sec7 domain-containing protein 1 (Iqsec1), x-ray repair cross complementing 4 (Xrcc4), homeodomain-interacting protein kinase 2 (Hipk2), and F-box/LRR repeat protein 5 (Fbxl5), respectively, which may be linked to neurocognitive disorders (Zhang et al., 2013; Gerez et al., 2019; Liang et al., 2020; Briševac et al., 2021). Moreover, circ_0001634 originates through back-splicing events from Fam53b, which has been reported to regulate Wnt signal transduction by altering β-catenin nuclear localization (Kizil et al., 2014). Wnt signaling has been implicated in the modulation of synaptogenesis, long-term potentiation (LTP), and dendrite arborization (Narvaes and Furini, 2022). Intranuclear accumulation of β-catenin marks activation of canonical Wnt signaling that sequentially leads to enhance the transcription of Wnt target genes via the interaction between β-catenin and T-cell factor/lymphoid enhancer factor (TCF/LEF) (Narvaes and Furini, 2022). Hu et al. (2016) found that prolonged exposure to 3.6% sevoflurane could disrupt BBB components via suppressing Wnt/β-catenin/Annexin A1 pathway in brain microvascular endothelial cells, indicating that promoting β-catenin synthesis can alleviate POCD. Hence, these five circRNAs were selected for verification. Quantitative RT-PCR array revealed that the augmented levels of circ_0001634, mmu_circ_0001345, and circ_0001493 were consistent with the sequencing results.

Based on ceRNA hypothesis that a protein-coding RNA and a non-coding RNA compete for the same miRNA through the shared miRNA response elements (MREs), miRNA serves as a bridge for circRNA-induced translation and/or stabilization of the mRNAs (Chen, 2020). In our study, miR-6912-5p, miR-490-5p, and miR-7001-5p were screened as hub nodes to compete with three circRNAs for governing the expression of six mRNAs. GO classification and enrichment analysis uncovered that cadherin 3 (Cdh3), secreted frizzled related protein 5 (Sfrp5), and sclerostin domain containing 1 (Sostdc1) in the ceRNA network participate in the regulation of the canonical Wnt signaling pathway. PCR analysis showed that anesthesia/surgery increased the expression of miR-6912-5p, RNA binding motif protein 47 (Rbm47), and Sostdc1 in the hippocampus, while decreased the expression of miR-490-5p, miR-7001-5p, Cdh3, and Sfrp5. Due to a positive expression correlation between genes and their corresponding circRNAs, circ_0001634/miR-490-5p/Rbm47, circ_0001634/miR-490-5p/Sostdc1, circ_0001634/miR-7001-5p /Sostdc1, circ_0001345/miR-7001-5p/Sostdc1, and circ_000149 3/miR-7001-5p/Sostdc1 axis might be existent in the pathogenesis of POCD. Pei et al. (2022) found that CircFAM53B, highly homologous to mmu_circ_0001634, impeded glioma cell apoptosis through sponging miR-532-3p, suggesting a potential role in neurological disorders. Interestingly, circFbxl5 (homologous with circ_0001345) could function as a miR-146a sponge in mouse cardiomyocyte (Li et al., 2022b), and miR-146a has been proven to ameliorate surgery-induced cognitive decline (Chen et al., 2019a). Accordingly, it is speculated that depletion of circ_0001345 may be an emerging therapeutic perspective on POCD. Most notably, miR-490-5p has been shown to ameliorate stroke-induced neurological dysfunction by repressing cyclin-dependent kinases 1 (CDK1) (Skovira et al., 2016; Ding et al., 2021), revealing a cognition-protective role for miR-490-5p. Rbm47, a central mediator of mRNA alternative splicing and stability, has been elaborated to suppress Wnt activity in cancer cells via maintaining AXIN1 or DKK1 mRNA stability (Vanharanta et al., 2014; Shen et al., 2020). Moreover, Sostdc1 has been documented to negatively modulate Wnt signaling (Li et al., 2022a). These imply that inhibition of Rbm47 or Sostdc1 may mitigate postoperative neurocognitive disorder.

Nonetheless, some limitations of this study must be considered. First, these three datasets are retrieved from different platform and samples, which certainly reduces confidence in the regulator-target pairs outlined. Second, the ideal scenario would be the same logFC threshold and adjusted *p*-value. However, adjusted *p*-value is either absent (GSE190880) or greater than 0.9 (GSE95070 and GSE115440), which may be attributed to the relative small sample size and unknown distribution of their genomic data. There are many DEcircRNAs but few DEmiRNAs in the case of *p* < 0.05 and |logFC| o > 1.0. Hence, *p*-value and different logFC thresholds were employed for screening DERNAs, as previously described (Wang et al., 2022). Third, limited RNA ID information of other datasets available such as GSE165798 (Wu et al., 2021) hampers the integration of multiple datasets. Finally, the circRNA-miRNA and miRNA-mRNA interaction relationships in the ceRNA network were both based upon a prediction algorithm that required further experimental verification.

In summary, a ceRNA regulatory network in the hippocampus of POCD mice included three circRNAs, three miRNAs, and six mRNAs. Through comprehensive bioinformatic analysis, the present study broadens our horizon on the occurrence and progression of POCD.

## Data availability statement

The original contributions presented in this study are included in the article/Supplementary material, further inquiries can be directed to the corresponding author/s.

## Ethics statement

The animal study was reviewed and approved by the Laboratory Animal Ethics Committee in Renmin Hospital of Wuhan University.

## Author contributions

WW, PH, LZ, GL, and ZX designed the study. WW and PH collected and analyzed the data. WW drafted the manuscript. GL and ZX undertook a critical revision of the manuscript. All authors contributed to the article and approved the submitted version.

## Footnotes

1
https://www.xiantao.love/

2
http://www.circbase.org

3
http://www.mirdb.org/

4
https://www.targetscan.org

5
https://www.kegg.jp

6
https://david.ncifcrf.gov/
